# Secondary Ammonium Agonists Make Dual Cation-π Interactions in α4β2 Nicotinic Receptors

**DOI:** 10.1523/ENEURO.0032-17.2017

**Published:** 2017-03-30

**Authors:** Michael R. Post, Gabrielle S. Tender, Henry A. Lester, Dennis A. Dougherty

**Affiliations:** 1 Division of Chemistry and Chemical Engineering, California Institute of Technology, Pasadena, CA 91125; 2 Division of Biology and Biological Engineering, California Institute of Technology, Pasadena, CA 91125

**Keywords:** Parkinson's disease, Addiction, Ion channels, Nicotinic acetylcholine receptors, Electrophysiology, Non-canonical amino acids

## Abstract

A cation-π interaction between the ammonium group of an agonist and a conserved tryptophan termed TrpB is a near universal feature of agonist binding to nicotinic acetylcholine receptors (nAChRs). TrpB is one of five residues that form the aromatic box of the agonist binding site, and for the prototype agonists ACh and nicotine, only TrpB makes a functional cation-π interaction. We report that, in addition to TrpB, a significant cation-π interaction is made to a second aromatic, TyrC2, by the agonists metanicotine, TC299423, varenicline, and nornicotine. A common structural feature of these agonists, and a distinction from ACh and nicotine, is a protonated secondary amine that provides the cation for the cation-π interaction. These results indicate a distinction in binding modes between agonists with subtly different structures that may provide guidance for the development of subtype-selective agonists of nAChRs.

## Significance Statement

The α4β2 nicotinic acetylcholine receptor (nAChR) binding site is made of several loops contributing five aromatic residues. Here, we show four secondary ammonium agonists, TC299423, metanicotine, varenicline, and nornicotine, make a cation-π interaction with TyrC2 in addition to the canonical cation-π interaction with TrpB. The prototypical agonists acetylcholine (a quaternary ammonium), and nicotine (a tertiary ammonium) only make a cation-π interaction with TrpB. This result indicates a new binding mode for agonists with only subtle structural differences and suggests that a more compact cation allows for greater interaction with loop C in the binding site.

## Introduction

**Figure 1.**
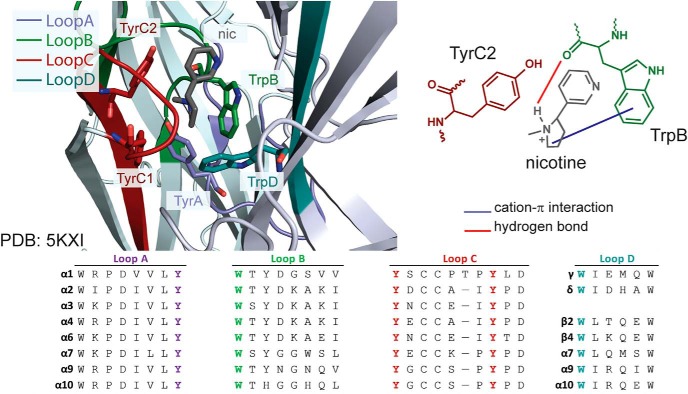
A view of nicotine at the α4β2 binding site. The crystal structure of α4β2 (PDB 5KXI) on the left shows the aromatic box motif, with each loop contributing to the binding site in a unique color and nicotine in gray. The schematic on the right details the hydrogen bond (red) and cation-π interaction (purple) interactions previously determined for nicotine with TrpB (α4: 149), as well as how TyrC2 (α4: 197) could interact with other agonists. TyrA (α4: 93), TyrC1(α4: 190), and TrpD (β2: 57) are shown in the crystal structure but omitted from the schematic for clarity. An alignment of each loop contributing to the box in the human nAChR family is shown at the bottom.

The neuronal nicotinic acetylcholine receptors (nAChRs) are members of the Cys-loop ligand-gated ion channel family and are established therapeutic targets for nicotine addiction, as well as possible targets for Parkinson’s disease, Alzheimer’s disease, pain, and other neural disorders (Romanelli et al., 2007). The receptors are pentamers, and 11 known subunits, α2-7,9,10 and β2-4, combine to form distinct subtypes (Le Novère et al., 2002; Millar, 2003; Gotti et al., 2006; Zoli et al., 2015). The nAChR binding site lies at the extracellular α-β interface, and it contains an aromatic box motif that binds the cationic moiety of the agonist through a cation-π interaction (Fig. 1; Dougherty, 1996, 2013; Van Arnam and Dougherty, 2014). Five aromatic residues are contributed by four loops, TyrA, Trp B, TyrC1, TyrC2, and TrpD (Corringer et al., 2000). In many studies of ligands binding to nAChRs, TrpB forms a functionally important cation-π interaction, while the other aromatics apparently play other roles (Xiu et al., 2009; Blum et al., 2010, 2013; Puskar et al., 2012; Tavares et al., 2012; Van Arnam and Dougherty, 2014).

A major goal in nAChR research is to develop agonists that target specific subtypes (Holladay et al., 1997; Quik and Wonnacott, 2011; Dineley et al., 2015). For example, the α4β2-containing subtypes are expressed throughout the brain and are most associated with several aspects of nicotine addiction (De Biasi and Dani, 2011). The α6β2-containing subtypes have a more restricted distribution. They occur on dopaminergic neurons, where they have been associated with reward-related behavior and Parkinson’s disease, as well as on medial habenula neurons, which play a role in aversive behavior (Quik and McIntosh, 2006; Jackson et al., 2013; Henderson et al., 2014; Zuo et al., 2016). Finding agonists that meaningfully distinguish between the α4β2 and α6β2 interfaces is an unsolved challenge, but metanicotine (rivanicline, TC-2403, or RJR-2403) and TC299423 have been found to preferentially activate α4β2- and α6β2-containing subtypes, respectively (Drenan et al., 2008; Grady et al., 2010; Xiao et al., 2011; Wall, 2015).

**Figure 2.**
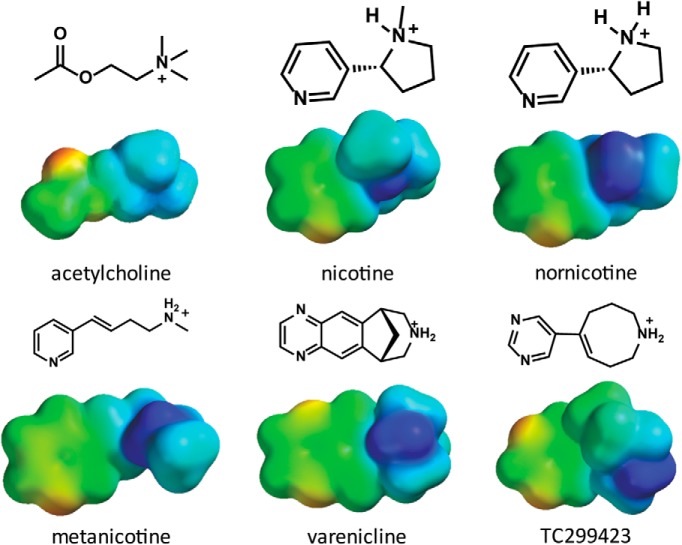
The structures and electrostatic potential maps of acetylcholine and nicotine are shown here for comparison to the secondary amine agonists and have been calculated with Hartree Fock 6-31G** (shown on a scale of −10, red and more negative electrostatic potential, to +150, blue and more positive electrostatic potential, kcal/mol).

Previous analysis of TC299423 at α6β2 showed an unusual binding pattern, in that the agonist does not make a functional cation-π interaction with TrpB or any other aromatic box residue (Post et al., 2015); thus, a unique binding mode may contribute to its subtype selectivity. Here, TC299423 and other agonists were studied at the more extensively characterized α4β2 receptor to see whether the unusual binding pattern persists. Several agonists were found to make cation-π interactions with both TrpB and TyrC2, and we show that this dual cation-π feature is a more general trend among secondary ammonium agonists (Fig. 2) at α4β2.

## Materials and Methods

### Molecular biology

Rat α4 and β2 subunits were used as the basis for the constructs. The L9’A mutation in the α4 M2 transmembrane domain, at the gate of the channel, was incorporated to amplify signal by shifting the stability of the channel partially toward the active state. This α4L’Aβ2 construct is described as wild type and/or α4β2 throughout the report for clarity in comparing noncanonical mutations made to the binding site, which is over 60 Å away from the channel gate. All constructs were in the pGEMhe vector, a cDNA plasmid optimized for protein expression in *Xenopus* oocytes. Site-directed mutagenesis was performed by PCR using the Stratagene QuikChange protocol, and primers ordered from Integrated DNA Technologies. Circular cDNA was linearized with SbfI (New England Biolabs) and then transcribed *in vitro* using T7 mMessage mMachine kit (Life Technologies), with a purification step after each process (QIAGEN). Final concentrations were quantified by UV spectroscopy.

### Ion channel expression

*Xenopus laevis* oocytes (stage V to VI) were sourced from both an institute facility and Ecocyte Bio Science. Oocytes were injected with 50-nL solution containing either 5- or 10-ng mRNA, injected in a 1:2 α4:β2 ratio to control for a pure population of the (α4L9’A)_2_(β2)_3_ stoichiometry. The alternative stoichiometry (α4)_3_(β2)_2_ has a much lower EC_50_ due to the extra L9’A mutation. We therefore avoided a mixed population containing both stoichiometries. Cells were incubated 24-48 h at 18°C in ND96 solution (96 mM NaCl, 2mM KCl, 1 mM MgCl_2_, and 5mM HEPES, pH 7.5) enriched with theophylline, sodium pyruvate, and gentamycin.

### Noncanonical amino acid incorporation

The cyanomethylester form of nitroveratryloxycarbonyl (NVOC)-protected tryptophan and phenylalanine analogues was coupled to dinucleotide dCA and enzymatically ligated to UAG-suppressor 74-mer THG73 tRNA_CUA_. The product was verified by Matrix-assisted laser desorption/ionization (MALDI) time-of-flight mass spectrometry on a 3-hydroxypicolinic acid matrix. The noncanonical amino acid-coupled tRNA was deprotected by photolysis either on a 500 W Hg/Xe arc lam, filtered with Schott WG-320 and UG-11 filters, or with an M365LP1 365 nm 1150 mW LED lamp (Thor Labs) immediately before coinjection with mRNA containing the UAG mutation at the site of interest. mRNA and tRNA were typically injected in a 1:1 or 1:2 volume ratio in a total volume of 50 or 75 nL, respectively, so that 25 ng of mRNA was injected per cell. In cases where observed agonist-induced currents were low after 48-h incubation, likely due to low protein expression, a second injection of mRNA and tRNA was performed after 24 h. The fidelity of noncanonical amino acid incorporation was confirmed at Trp with a wild-type recovery experiment where tryptophan was loaded onto tRNA. If this experiment yielded similar to EC_50_ to wild type, then the cell incorporated the charged residue and nothing else. This was accomplished with the Tyr sites by comparing tRNA charged with Phe to a conventional Tyr-Phe mutation. A read-through/reaminoacylation test served as a negative control by injecting unacylated full-length 76-mer tRNA. Lack of current proved no detectable reaminoacylation at the suppression site.

### Whole-cell electrophysiological characterization

(S)-nornicotine hydrochloride was purchased from Matrix Scientific, while varenicline (Pfizer), metanicotine, and TC299423 (Targacept) were generous gifts. Agonist-induced currents were recorded in TEVC mode using the OpusXpress 6000A (Molecular Devices) at a holding potential of −60 mV in a running buffer of Ca^2+^-free ND96, which since α4β2 is Ca^2+^ permeable, prevents interference from Ca^2+^-activated channels endogenous to the oocyte. Agonists were prepared in Ca^2+^-free ND96 and delivered to cells via a 1-mL application over 15 s followed by a 2-min wash. For data from dose-response experiments were normalized, averaged, and fit to the Hill equation using Kaleidagraph (Synergy Software). In data tables, *N* is the total number of oocytes analyzed, and cells from different frogs on at least two different days were used for each point. Fluorination plots are visualized here with Prism (GraphPad Software). EC_50_ and Hill coefficient errors are presented as SEM.

## Results

### Binding studies of TC299423 and metanicotine at α4β2

All studies here used the previously described (αL9’A)_2_(β2)_3_ receptor (Kuryatov et al., 2005; Nelson et al., 2003). TC299423 was first probed for cation-π interactions at TrpB and TyrC2 (TyrA, TyrC1, and TrpD have never been implicated in a cation-π interaction). In these experiments, the site of the aromatic residue of interest is mutated to a TAG stop codon. mRNA made *in vitro* is injected into *Xenopus* oocytes alongside a bioorthogonal tRNA_CUA_ that has been chemically appended to the noncanonical amino acid of interest. To probe for an agonist cation-π interaction, a series of residues with electron-withdrawing groups that weaken the interaction is used. Typically, fluorotryptophans (F_n_Trp) are used to probe Trp and fluorophenylalanines (F_n_Phe) are used to probe Tyr (fluorinating tyrosine causes the phenol group to deprotonate at physiologic pH). The endpoints of the two series, F_4_Trp and F_3_Phe, are both thought to approximate a situation in which the dominant electrostatic component of the cation-π interaction has been completely removed, allowing a semiquantitative comparison of Trp and Tyr residues. Any change in binding is revealed by a changed EC_50_ value, monitored by two-electrode voltage clamp electrophysiology dose-response experiments. If the interaction is weakened by these substitutions, EC_50_ correspondingly increases. This change is visualized in so-called fluorination plots of the log of the fold-shift in EC_50_ against the calculated gas-phase cation-π interaction strength.

**Figure 3.**
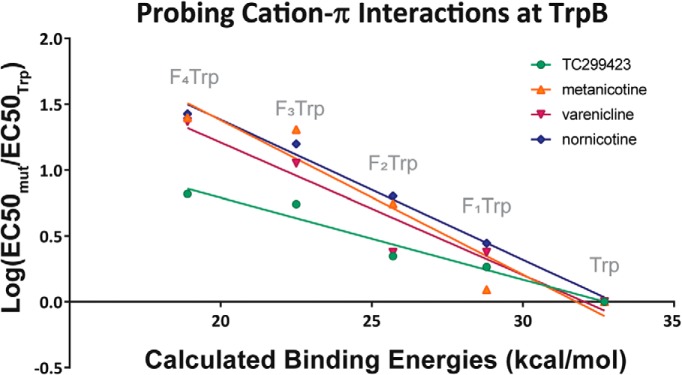
Fluorination plots of all the agonists tested in this report at TrpB in α4β2. The *x*-axis is the predicted M06/6-31G(d,p) DFT-calculated energies between a sodium ion and each side chain (labeled) in the gas phase as described in Davis and Dougherty (2015). The *y*-axis is the log of the fold-shift in EC_50_. Each agonist tested showed a linear trend and, therefore, demonstrated a functional cation-π interaction at TrpB, as previously seen with acetylcholine and nicotine. Data plotted for varenicline are from Tavares et al. (2012).

At TrpB, TC299423 showed an increase in EC_50_ with each additional fluorine substituent on the ring (Table 1), but the maximum fold-shift in EC_50_ observed at F_4_Trp was only 6.6-fold. While this is a modest loss of function, ACh experiences a 66-fold loss of function at F_4_Trp in α4β2 (Xiu et al., 2009), there is nevertheless a linear trend in the fluorination plot (Fig. 3). Thus, it can be said that TC299423 makes a functional, if modest, cation-π interaction with TrpB in α4β2.

**Table 1:** TC299423

TrpB	EC_50_ (μM)	n_H_	I_max_ (μA)	Fold shift	*N*
Trp	0.023 ± 0.0009	1.4 ± 0.06	0.22–1.46	1	15
F_1_Trp	0.043 ± 0.0008	1.3 ± 0.03	0.12–1.2	1.8	11
F_2_Trp	0.052 ± 0.001	1.2 ± 0.04	0.05–0.62	2.2	14
F_3_Trp	0.13 ± 0.003	1.2 ± 0.03	0.11–1.41	5.5	12
F_4_Trp	0.15 ± 0.007	1.1 ± 0.05	0.15–0.77	6.6	14
TyrC2	EC_50_ (μM)	n_H_	I_max_ (μA)	Fold shift	*N*
Phe	0.098 ± 0.003	1.1 ± 0.03	0.06–1.08	1	17
F_1_Phe	0.14 ± 0.005	1.2 ± 0.04	0.05–0.38	1.5	12
F_2_Phe	1.6 ± 0.07	1.3 ± 0.06	0.05–0.57	16	9
F_3_Phe	3.0 ± 0.25	1.3 ± 0.11	0.07–0.18	30	7

**Figure 4.**
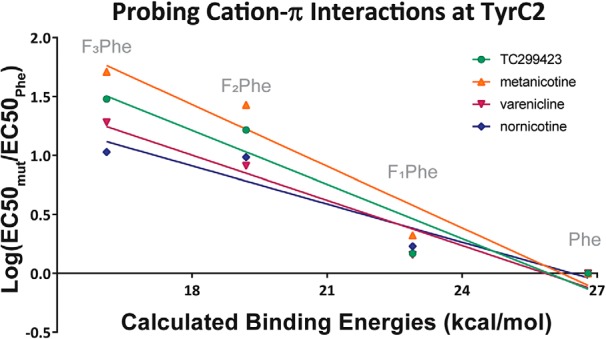
Fluorination plots of all the agonists tested in this report at TyrC2 in α4β2. The *x*-axis is the predicted M06/6-31G(d,p) DFT-calculated energies between a sodium ion and each side chain (labeled) in the gas phase as described in Davis and Dougherty (2015). The *y*-axis is the log of the fold-shift in EC_50_. Each agonist tested shows a linear trend and, therefore, demonstrates a functional cation-π interaction with TyrC2, a result not previously seen with acetylcholine or nicotine.

TyrC2 was then probed for a cation-π interaction with TC299423 and showed an unexpected trend, with F_3_Phe substitution resulting in a 30-fold increase in EC_50_. When presented as a fluorination plot (Fig. 4), these results show a linear trend, showing that in addition to a cation-π interaction with TrpB, TC299423 makes a functional, and energetically more significant, cation-π interaction at TyrC2.

Metanicotine, an isomer of nicotine in which the pyrrolidine ring has been opened, has antinociceptive effects in mice and is more potent and efficacious than ACh at α4β2 receptors (Damaj et al., 1999; Papke et al., 2000). When analyzed via a fluorination series at TrpB in α4β2, metanicotine displayed a functional cation-π interaction, with a linear fluorination plot and F_4_Trp resulting in a 25-fold shift in EC_50_ (Table 2; Fig. 3). TyrC2 also shows a linear fluorination plot, with the F_3_Phe mutation causing a 51-fold shift relative to Phe (Fig. 4). TyrA was probed and showed no meaningful changes in metanicotine EC_50_ on fluorination (Table 2; nicotine and ACh also showed no meaningful shifts in EC_50_ at this site; Xiu et al., 2009). Thus, metanicotine also forms dual, functional cation-π interactions at TrpB and TyrC2 in α4β2.

**Table 2:** Metanicotine

TrpB	EC_50_ (μM)	n_H_	I_max_ (μA)	Fold shift	*N*
Trp	0.64 ± 0.02	1.3 ± 0.0	0.08–0.89	1	13
F_1_Trp	0.79 ± 0.02	1.4 ± 0.0	0.12–0.80	1.2	16
F_2_Trp	3.6 ± 0.1	1.5 ± 0.1	0.15–0.56	5.6	14
F_3_Trp	13 ± 1	1.6 ± 0.1	0.07–0.30	20	12
F_4_Trp	16 ± 2	1.3 ± 0.1	0.03–0.11	25	12
TyrA	EC_50_ (μM)	n_H_	I_max_ (μA)	Fold shift	*N*
Phe	19 ± 5	1.1 ± 0.2	0.02–0.16	1	7
F_3_Phe	17 ± 2	1.3 ± 0.1	0.01–0.03	0.9	6
TyrC2	EC_50_ (μM)	n_H_	I_max_ (μA)	Fold shift	*N*
Phe	0.41 ± 0.03	1.2 ± 0.07	0.06–2.78	1	17
F_1_Phe	0.86 ± 0.06	1.2 ± 0.08	0.04–0.07	2.1	11
F_2_Phe	11 ± 1	0.6 ± 0.1	0.03–0.13	27	8
F_3_Phe	21 ± 1	1.4 ± 0.2	0.04–0.24	51	12

Both metanicotine and TC299423 are typical nicotinic pharmacophores in that they have a cationic amine moiety, a hydrogen bond donor associated with that amine, and a hydrogen bond acceptor several angstroms away (Blum et al., 2010). In contrast to the tertiary ammonium nicotine and the quaternary ammonium ACh, metanicotine and TC299423 are both secondary ammonium ions. Thus, to test whether this feature was associated with the novel dual cation-π interaction, additional secondary amine agonists were analyzed.

### Establishing a binding trend for secondary amines

Varenicline (Chantix) is a smoking cessation drug that is thought to work by serving as a partial agonist to α4β2 (Coe et al., 2005a, 2005b) It has a secondary ammonium as its cationic center. This drug has previously been shown to form a cation-π interaction at TrpB in α4β2 (Tavares et al., 2012), with a 23-fold shift in EC_50_ at F_4_Trp (Table 3; Fig. 3), but had not been analyzed at TyrC2.

**Table 3:** Varenicline

TrpB*	EC_50_ (μM)	n_H_	Fold shift	*N*
Trp	0.0024 ± 0.0001	1.2 ± 0.1	1	15
F_1_Trp	0.0057 ± 0.0002	1.2 ± 0.1	2.4	11
F_2_Trp	0.0057 ± 0.0021	1.2 ± 0.1	2.4	14
F_3_Trp	0.027 ± 0.001	1.3 ± 0.1	11	12
F_4_Trp	0.056 ± 0.005	1.1 ± 0.1	23	14

TyrC2	EC_50_ (μM)	n_H_	I_max_ (μA)	Fold shift	*N*
Phe	0.0014 ± 0.0002	1.3 ± 0.14	0.04–0.14	1	10
F_1_Phe	0.0020 ± 0.00009	1.5 ± 0.09	0.02–0.08	1.4	8
F_2_Phe	0.011 ± 0.00097	1.2 ± 0.11	0.02–0.1	8.1	12
F_3_Phe	0.027 ± 0.0016	1.1 ± 0.06	0.02–0.09	19	8

Nonsense-suppression-based fluorination studies were conducted for varenicline at TyrC2 as discussed above. The corresponding fluorination plot shows a linear trend with a 19-fold shift for F_3_Phe, confirming that varenicline makes a cation-π interaction with TyrC2 in α4β2 (Table 3; Fig. 4).

Varenicline was the third secondary ammonium agonist to demonstrate functional cation-π interactions with both TrpB and TyrC2 in α4β2. To support the notion that a dual cation-π interaction is associated with secondary ammonium agonists in α4β2, we transformed nicotine, which makes a single cation-π interaction at TrpB, into a secondary ammonium. Nornicotine, nicotine that has been demethylated at the pyrrolidine N, is a natural component of tobacco that is a precursor to the well-documented carcinogen *N*’-nitrosonicotine that is a by-product of the curing process.(Siminszky et al., 2005)

**Figure 5.**
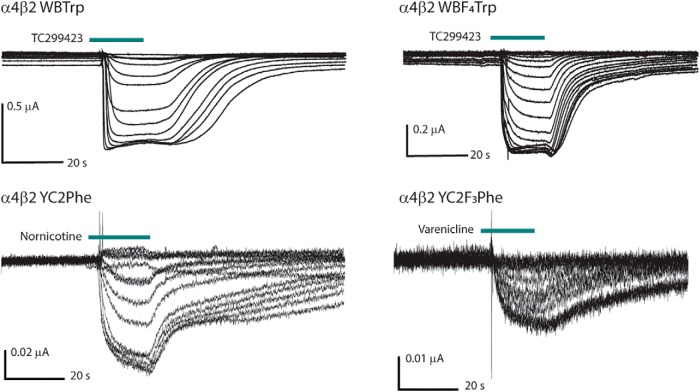
Representative traces from dose-response experiments with a variety of agonists, noncanonical amino acid substitutions, and I_max_ values.

Nornicotine is much less potent than its methylated analog, with an EC_50_ of 1.8 μM, a 20-fold greater value than for nicotine. This agonist also elicits much smaller currents; however, the waveform shapes (Fig. 5) are consistent with other agonists, and a clear dose-response relation is present. The fluorination plot of nornicotine at TrpB shows a cation-π interaction, with F_4_Trp resulting in a 27-fold shift in EC_50_, demonstrating a functionally important cation-π interaction (Table 4; Fig. 3). Results at TyrC2 show the same type of trend seen for other secondary ammonium agonists analyzed in this report, with a linear fluorination plot and a 11-fold loss of function for F_3_Phe (Table 4; Fig. 4).

**Table 4:** Nornicotine

TrpB	EC_50_ (μM)	n_H_	I_max_ (μA)	Fold shift	*N*
Trp	1.7 ± 0.1	1.3 ± 0.1	1.33–9.37	1	13
F_1_Trp	4.6 ± 0.2	1.3 ± 0.1	0.27–0.9	2.8	16
F_2_Trp	11 ± 0.7	1.3 ± 0.1	0.04–0.11	6.4	8
F_3_Trp	26 ± 2	1.5 ± 0.1	0.05–1.28	16	16
F_4_Trp	44 ± 4	1.2 ± 0.1	0.95–1.51	27	8
TyrC2	EC_50_ (μM)	n_H_	I_max_ (μA)	Fold shift	*N*
Phe	3.3 ± 0.3	1.2 ± 0.1	0.02–1.42	1	15
F_1_Phe	5.5 ± 0.3	1.0 ± 0.1	0.04–0.11	1.7	11
F_2_Phe	31 ± 3	1.2 ± 0.1	0.03–0.26	9.6	10
F_3_Phe	35 ± 2	1.4 ± 0.1	0.02–0.14	11	12

## Discussion

Structure-function studies of four different agonists with distinct overall structures but a common secondary ammonium moiety have established a functional cation-π interaction with both TrpB and TyrC2 in α4β2 nAChRs. Nornicotine forms a cation-π interaction with TyrC2, but nicotine, which only differs from nornicotine by being a tertiary rather than secondary ammonium, does not. This nicotine/nornicotine comparison in particular presents a compelling case that the dual cation-π interaction is a consequence of the secondary ammonium group of select agonists at α4β2.

The significance of TrpB in agonist binding to nAChRs remains a central tenet of the pharmacology of this system. In a cation-π interaction, the aromatic ring of Trp is a stronger binding site that those of Tyr or Phe, regardless of the nature of the cation, but Tyr and Phe can certainly make strong cation-π interactions (Davis and Dougherty, 2015). Early studies focused on ACh and nicotine and found that only TrpB showed a strong response to fluorination. We have now found that four other agonists show, in addition to TrpB, a significant response to fluorination at TyrC2. These four are structurally diverse but share a common feature of being a secondary ammonium. The implication is clear that the more compact secondary ammonium is able to establish an additional interaction compared with the bulkier quaternary (ACh) or tertiary (nicotine) systems.

Two studies of the primary ammonium agonist GABA at pentameric receptors, one at the RDL insect GABA receptor and one at the prokaryotic *Erwinia* ligand-gated ion channel (ELIC) receptor, show that this primary ammonium agonist makes functionally important cation-π interactions to the aromatics at positions B and C2 (Lummis et al., 2005; Spurny et al., 2012). Again, a more compact agonist can make a dual cation-π interaction. A recent computational study of the AChBP aromatic box suggests that the side chains of each aromatic box residue can contribute to the overall cation-π binding energy in the ACh-AChBP complex (Davis and Dougherty, 2015). However, from a functional perspective, only TrpB is universally important, with TyrC2 being identified here as contributing in some, but not all, cases.

A popular model for nAChR gating proposes that loop C moves on agonist binding so as to clamp down on the agonist and more clearly define the aromatic box (Wang et al., 2009). This movement of loop C is proposed to be a key functional feature of the gating mechanism. It may be that with the less bulky secondary ammonium agonists, loop C is able to move closer to the agonist. This larger motion by loop C leads to a closer contact between TyrC2 and the agonist, enabling a cation-π interaction and making TyrC2 responsive to fluorination. AChBP structures with varenicline versus nicotine bound do not show a meaningful difference in the position of loop C, but AChBP did not evolve to undergo a gating process and likely undergoes minimal conformational changes when binding small molecules (Celie et al., 2004; Rucktooa et al., 2012).

In summary, we have found a distinction in the binding mode of agonists at the α4β2 nAChR. The natural agonist ACh and the prominent component of tobacco nicotine both make a cation-π interaction to TrpB, along with other hydrogen bonding interactions. In contrast, four agonists that share a common feature of being secondary ammonium ions make a dual cation-π interaction to TrpB and TyrC2. This pattern may be unique to the α4β2 subtype, as it was not reported for TC299423 at the α6β2 subtype (Post et al., 2015). Further studies of other agonists and other subtypes could provide valuable guidance in designing more subtype-selective activators of nAChRs.

## Synthesis

Reviewing Editor: Robert Kalb, Children's Hospital of Philadelphia Decisions are customarily a result of the Reviewing Editor and the peer reviewers coming together and discussing their recommendations until a consensus is reached. When revisions are invited, a fact-based synthesis statement explaining their decision and outlining what is needed to prepare a revision will be listed below. The following reviewer(s) agreed to reveal their identity: Pierre-Jean Corringer, Jon Lindstrom

Both reviewers were enthusiastic about this work. Reviewer #1 states that “The conclusion is also important, since it is shown that nicotinic ligands carrying a secondary ammonium make cation-pi interaction not only with the Trp from loop B, but also with a tyrosine from loop C that is not normally engaged in such type of interaction with nicotine and acetylcholine. The beauty of the approach is that these molecular details are investigated on the functional receptor and are thus directly relevant to its physiological regulation. This observation will be useful for design of new nicotinic ligands. ” . Reviewer #2 states that the work is elegant and that “ that tyrosine C2 forms a cation-pi interaction with varenicline and some other agonists, but not ACh or nicotine, as a consequence of the secondary ammonium group of varenicline etc. All of these agonists form cation-pi interactions with tryptophan B. The significance of this second cation-pi interaction in the affinities pr potencies of these various agonists is not clear, though it is suggested that it might contribute to selectivity differences between alpha 4/beta 2 and alpha 6/beta 2 ACh sites. ”

There were a number of issues raised and the authors need to address them:

- Figure 1 could be made clearer, especially for non-specialists. The left panel should show a bound ligand, and its pose related to the aromatic box. ideally, an AChBP structure in complex with nicotine and other ligands could be included, to see the atomic details of the orthosteric site. A sequence of the C-loop would be useful to locate the tyrosine.

- At the end of the introduction, it is stated that the aim of the study was to explain the preference of TC299423 for activating alpha6beta2 nAChR. It is found here that TC299423 make cation-pi interaction only with alpha4beta2 receptor. Therefore, the preference for the alpha6 receptor remains elusive to me.

- At the start of the result section, authors should explain why they use the (αL9'A)2(β2)3 receptor to conduct their study.

- In the various tables, the Imax of many mutants appears to by very low (as low as 30 nA). Therefore, representative trace currents of the various mutants should be systematically included to allow the reader to evaluate the significance of the observation

- In the discussion, it is stated that a more compact secondary ammonium is necessary to make cation pi interaction with loop C tyrosine. Is the compactness really the reason for the observation? This is only one possibility. Would it be possible that secondary ammonium preferentially interact with phenyl/phenol groups as compared to indole, among other possibilities ?

- Author should also show and/or discuss the conservation of the loopC Tyr among nAChR subtypes.

- On page 4 the mutation alpha 4 L9'A is described as “wild type” or “alpha 4”. Presumably this means that the alpha 4 L9'A mutation in the M2 transmembrane domain was used to weaken the channel gate and thus amplify responses. It would be better to clearly say what they mean.

- Oocytes were studied in calcium free medium. They should clearly say why this was done.

- They inject subunit mRNA at a 1:2 alpha 4:beta 2 ratio. They should say that this was to promote assembly of the (alpha 4 beta 2)2 beta 2 stoichiometry. Substantial amounts of the (alpha 4 beta 2)2 alpha 4 stoichiometry with a third alpha 4/alpha 4 ACh site would have compromised their functional assays.
